# Toward therapnostic integration as a new paradigm for precision management of interstitial cystitis driven by medical-engineering convergence

**DOI:** 10.1186/s11671-026-04857-3

**Published:** 2026-08-10

**Authors:** Weilun Zhang, Minli Shi, Yuxin Zhang, Weibing Shuang, Lei Pang

**Affiliations:** 1 Department of Urology, The Fifth Hospital of Shanxi Medical University (Shanxi Provincial People’s Hospital), Shanxi, 030012 China; 2 Department of Urology, The First Hospital of Shanxi Medical University, Shanxi, 030012 China

**Keywords:** Interstitial cystitis, Medical-engineering integration, Intelligent materials, Precision medicine, Nanomedicine, Digital twin

## Abstract

Interstitial Cystitis/Bladder Pain Syndrome (IC/BPS) is a complex chronic inflammatory disease involving mucosal barrier defects, neurogenic inflammation, mast cell activation, and tissue remodeling. Current diagnosis lacks objective biomarkers, relying instead on symptom assessment and exclusion of confusable diseases. Cystoscopy is recommended for identifying Hunner lesions per ESSIC/AUA guidelines, and first-line intravesical GAG replacement therapies only achieve sustained response rates of 20–38% at 12 months. Medical-engineering integration and materials innovations propel IC management toward multi-scale regulation and intelligent repair. This article proposes intelligent materials and systems to advance IC from stepwise diagnosis to an integrated "sensing–feedback–execution" closed loop. At the diagnostic level, it integrates medical phenotyping with engineering multidimensional detection to construct a synergistic system of subjective symptoms, objective molecules, and physical function: attomolar long non-coding RNA(lncRNA) detection via CRISPR-graphene transistors, glycosaminoglycans (GAGs) quantification with ^13^C-FNDs, and real-time bladder monitoring with flexible electrodes and wireless sensing. At the therapeutic level, centering on targeted delivery, dynamic repair, functional remodeling, it integrates four pathways: inflammation-responsive engineered bacteria; DNA nanocages and dual-responsive hydrogels for precise release; bioprinted patches and digital twin scaffolds for personalized repair; and trimodal combination therapy for mucosa, inflammation, and fibrosis. It further envisions Material Intelligence Agent systems medicine integrating nanosensors, quantum computing, and micro-nanorobots to construct a cross-scale diagnostic and therapeutic closed loop, with dynamic individualized intervention via digital twins. This paper provides a systematic technological pathway for precision IC management and a transferable medical-engineering integration paradigm for chronic inflammatory diseases.

## Introduction

IC/BPS is a chronic inflammatory condition with a complex etiology. Its diagnosis lacks objective biomarkers and relies on subjective symptoms and invasive cystoscopy, presenting significant challenges to conventional diagnostic and therapeutic paradigms. Research indicates that smart materials, capable of perceiving environmental stimuli (such as pH, enzymes, and mechanical stress) and executing specific responses, offer novel pathways to address the therapeutic challenges of IC [1]. The concept of systems emphasizes the integration of these materials to construct closed-loop frameworks with sensing-feedback-execution capabilities, enabling dynamic and precise management of the disease. This article prospectively examines various intelligent material systems—including nanosensors, bioresponsive hydrogels, engineered living cells, and flexible electronic devices—and explores their potential application pathways in the diagnosis and treatment of IC. These systems encompass multiple spatial scales—ranging from the molecular and cellular to the tissue and organ levels—while integrating diverse functional dimensions, including diagnosis, therapy, and monitoring. Collectively, they constitute a "multidimensional intelligent diagnostic and therapeutic system" tailored for the precision management of IC. This article aims to elucidate how intelligent materials and systems can drive transformative advances in the IC diagnostic and therapeutic paradigm. This article will first delineate how the integration of medical phenotyping with engineering detection technologies, such as quantum dot-based exosome sensing and CRISPR-powered graphene transistors, can facilitate a multi-dimensional precision identification system for IC. Second, it will explore how bio-hybrid systems, including engineered bacteria and responsive hydrogels, can execute targeted therapeutic tasks, such as inflammation regulation and barrier repair. Third, it will prospect the application of biomimetic manufacturing and organoid intelligence in tissue engineering therapy for bladder regeneration and functional monitoring. Finally, it will construct a vision for cross-domain neuromodulation and systems medicine, integrating flexible electronics and AI agents, to outline a future pathway toward comprehensive disease management.

### Diagnostic innovation: medical-engineering integration drives a multi-dimensional precision identification system for IC

#### Traditional diagnostic predicaments and the transformative role of medical-engineering integration

The diagnosis of IC/BPS has long relied on subjective symptom assessment and invasive cystoscopy, lacking objective molecular markers [2]. This traditional approach, while clinically useful, suffers from significant limitations, including the absence of biomarkers for early detection, reliance on morphological observation without functional quantification, and poor patient tolerability of repeated invasive procedures. Medical-engineering integration offers a paradigm shift toward multidimensional precision identification that addresses these gaps.

IC presents distinct molecular and microenvironmental signatures—exosomal integrin α_3_β_1_, GAG layer defects, inflammatory lncRNAs, epigenetic dysregulation, and acidic pH [3]. By coupling clinical phenotyping with cutting-edge engineering tools, IC diagnosis can transition from subjective judgment to objective quantification across molecular, physical, and functional dimensions. Specifically, we envision several complementary technological platforms: topological insulator quantum dot–enhanced mass spectrometry for ultrasensitive detection of urinary exosomal integrin α_3_β_1_ [4–7]; ^13^C-enriched fluorescent nanodiamonds (^13^C-FNDs) for dynamic assessment of glycosaminoglycan (GAG) layer status [8, 9]; methylation-sensitive DNA nanomachines for in situ epigenetic sensing of HDAC and DNMT activity [10–13], DNA origami-based enzyme detection and G-quadruplex readouts have been validated individually, but their integration into a single nanomachine for multiplexed epigenetic detection in clinical IC specimens remains a conceptual proposal requiring further validation [14]; and CRISPR–graphene field-effect transistors for attomolar-level detection of inflammatory long non-coding RNAs [15]. These technologies collectively form a synergistic "symptom–molecule–function" diagnostic system that integrates medical phenotyping with engineering precision, enabling earlier detection, more accurate subtyping, and direct therapeutic targeting. To enable a structured comparison of these emerging diagnostic platforms, we present their key features in Table 1. The following subsections detail each diagnostic platform and their collaborative integration.

**Table 1:** Molecular diagnostic technologies for IC

Molecular diagnostic technology	Detection targets	Detection sensitivity	Technical maturity	Validation status	Potential application scenarios in IC
CRISPR-graphene field-effect transistors (CRISPR-GFETs)	Inflammation-related long non-coding RNAs (lncRNAs)	Attomolar (aM) level	Laboratory R&D stage, preclinical validation ongoing	spiked urine samples; clinical validation in IC patient cohorts ongoing	Early screening of IC, quantification of inflammatory activity, primary screening alternative to some invasive examinations
Topological insulator quantum dots (Bi₂Se_3_ QDs) coupled with mass spectrometry	Urothelial exosomal integrin α_3_β_1_	Ultrasensitive (single-molecule level)	Prototype validation, small-scale clinical exploration ongoing	in exosome isolates from urine samples	Molecular phenotyping of IC, detection of lesion-specific biomarkers
^13^C-enriched fluorescent nanodiamond probes (^13^C-FNDs)	Glycosaminoglycan (GAG) layer metabolism/defect extent	Quantitative visualization with high spatial resolution	Laboratory imaging stage, compatible with endoscopic combined detection	in ex vivo bladder tissue; in vivo imaging studies ongoing in animal models	Subtyping of Hunner lesion IC, dynamic monitoring of GAG layer repair efficacy
Methylation-sensitive DNA nanomachines (MSNM)	HDAC activity, DNMT3B activation status	In situ highly specific detection	Basic research stage, compatible with tissue biopsy	in cell lines and fresh frozen biopsy samples	Confirmation of Hunner lesion-type IC, differential diagnosis of epigenetic subtypes

### Medical-engineering collaborative diagnosis: constructing a comprehensive diagnostic system of subjective symptoms-objective analysis-physical function

Medical diagnosis focuses on clinical phenotypes and pathological features, while engineering diagnosis emphasizes molecular identification and dynamic monitoring. Their deep integration forms a three-dimensional collaborative framework that significantly improves diagnostic accuracy, sensitivity, and early detection capacity in IC.

Medical–engineering collaborative diagnosis: Using typical IC symptoms as the initial screening criteria, simultaneously employing the Bi_2_Se_3_ topological insulator quantum dot (Bi_2_Se_3_ QDs) modified microfluidic mass spectrometry platform to detect urothelial exosomal integrin α_3_β_1_, and CRISPR-actuated graphene field-effect transistors (*Model predictions*) to detect inflammation-related lncRNA. It is suggested to conduct small-sample clinical validation (n ≥ 50) to assess the consistency between engineering indicators and cystoscopy results, then gradually explore the possibility of replacing the gold standard. If both engineering indicators are positive and symptoms meet the criteria, a preliminary diagnosis could be made without immediate invasive cystoscopy. Given the ultrasensitive detection capacity of CRISPR–graphene biosensors for low-abundance nucleic acids, we propose that CA-GFETs could achieve early detection of IC risk well before abnormalities become detectable by conventional urodynamic assessments. This would both shorten the diagnostic cycle and improve sensitivity. See Table 2 for current performance benchmarks and limitations.

**Table 2:** Current benchmarks, limitations, and future perspectives of emerging technologies for IC/BPS

Technology platform	Current benchmarks (what has been demonstrated)	Key limitations in IC/BPS (Gaps to address)	Future vision / proposed integration
CRISPR-GFETs	Attomolar (aM) sensitivity for nucleic acids in buffer; fM detection in urological disease models	Signal reduced 2–3 orders of magnitude in urine due to matrix interference; no IC/BPS cohort validation yet	Non-invasive urinary lncRNA screening as triage tool before cystoscopy
^13^C-FNDs	Millimeter-scale spatial resolution in hyperpolarized MRI; nanoscale resolution in cellular thermometry; excellent photostability	Quantitative correlation with histologically measured GAG thickness not reported; in vivo human bladder imaging not validated	Intraoperative fluorescence–MRI dual-modal imaging for real-time GAG defect mapping
Engineered EcN	Localized anti-inflammatory cytokine delivery in IBD models; quorum-sensing circuits functional	> 99% washout within 2–4 h in rodent bladder; safety in urothelium unproven; no IC/BPS model validation	Co-instillation with thermosensitive hydrogels for sustained bladder residence and on-demand secretion
DNA nanocages (TDN)	pH-dependent cargo release in buffer (tenfold increase at pH 6.0 vs. pH 7.4) [22]; programmable targeting and dual-drug loading	Nuclease degradation in urine not systematically quantified; penetration efficiency across GAG layer unknown	Acidic urine-triggered delivery of epigenetic modulators and anti-fibrotic agents
Magnetoelectric nanorobots	Fe_3_O_4_/BaTiO_3_ core–shell interfacial structure characterized at nanoscale; magnetoelectric coupling demonstrated in materials science	No bladder epithelial permeability data; magnetic field penetration and safety in pelvis unquantified	External alternating magnetic field-guided deep tissue drug penetration across urothelial barrier
Thermoresponsive hydrogels	> 24 h intravesical retention in rat models; 35% improvement in bladder compliance and 50% reduction in inflammatory infiltration in cystitis models	Mechanical fatigue and detachment after 10–20 filling-voiding cycles; long-term biocompatibility with repeated instillations unevaluated	4D bioprinted patches with patient-specific digital twin-guided ECM-mimetic architecture
GM-FEA	Stable impedance (~ 585 kΩ) and low noise for 45 days in *vivo*; simultaneous electrophysiology and calcium imaging demonstrated	No implantation or chronic recording reported in pelvic neural anatomy; effects of bladder deformation on electrode stability unknown	Closed-loop sacral neuromodulation with real-time pathological afferent signal detection
PENG	Validated for energy harvesting in cardiovascular and gastrointestinal systems; BaTiO_3_ nanowire arrays generate 1–10 V under 5–10% strain	Not tested under urodynamic conditions in bladder; actual power density from human bladder contractions unknown	Self-powered implantable neuromodulation devices harvesting energy from natural bladder wall motion

Invasive Cystoscopy and Optical Imaging Synergy for Subtyping: During cystoscopic observation of Hunner's lesions, ^13^C-FNDs enable fluorescence–NMR dual-modal imaging to quantify GAG layer defects, while quantum dot fluorescence detects Claudin- 4 expression. This approach refines lesion subtyping and provides a precise molecular basis for targeted GAG supplementation therapy in severe IC, enabling simultaneous identification of morphological subtype and therapeutic target. Quantitative validation status of ^13^C-FNDs is summarized in Table 2.

**Fig. 1:**
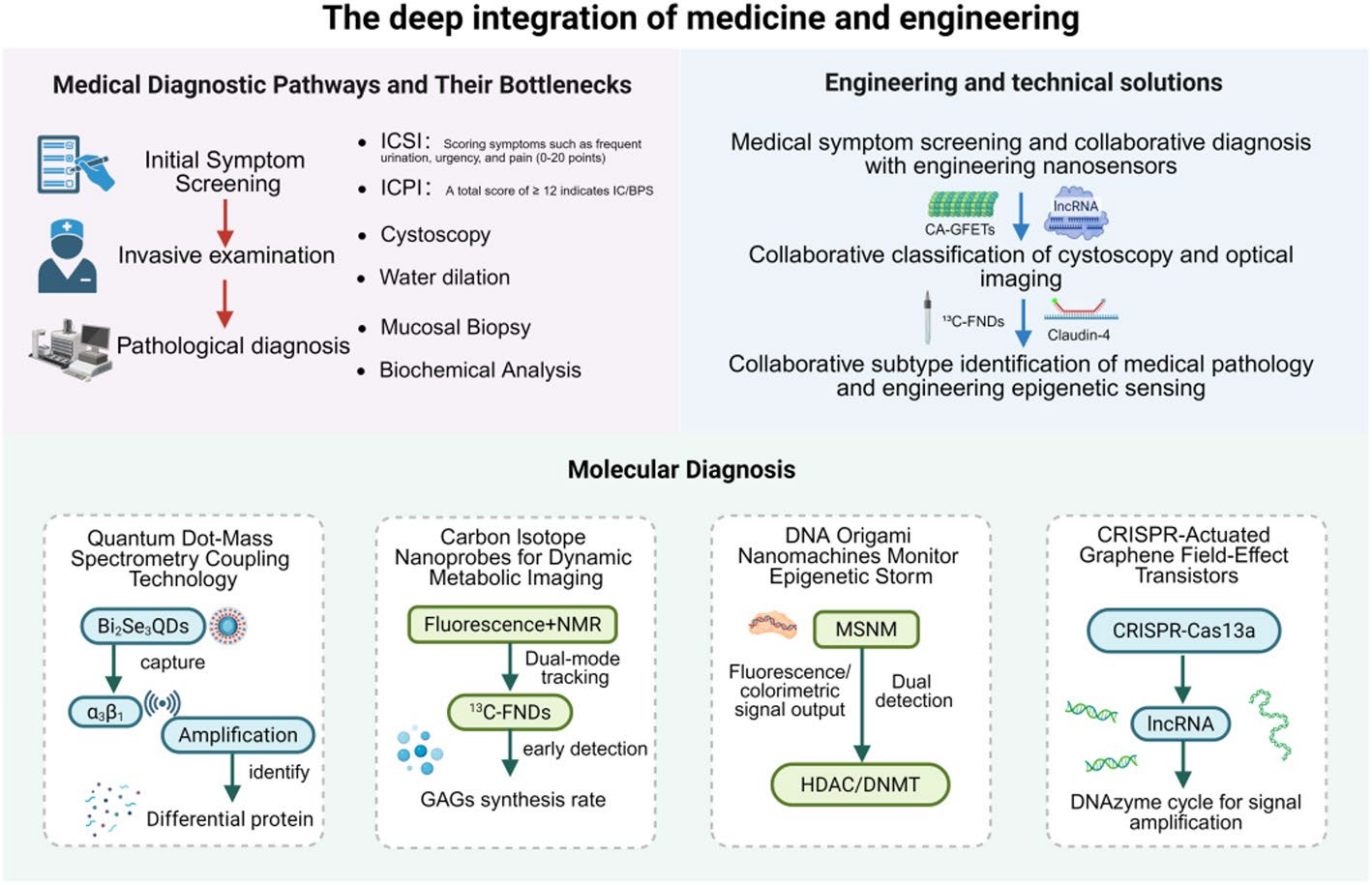
Schematic diagram of molecular diagnostic technologies for IC

Medical Pathology and Engineering Epigenetic Sensing Synergy for Subtype Differentiation: Performing both pathological sectioning and MSNM detection of HDAC activity and DNMT3B activation status simultaneously on biopsy tissue. Positive results for both can further confirm Hunner's ulcer type IC, enhancing differentiation accuracy while providing molecular-level support for the selection of individualized treatment plans such as endoscopic ablation (Fig. 1).

## Therapeutic breakthrough: focusing on treatment breakthroughs for IC

The pathological mechanisms of IC involve multidimensional issues, including mucosal barrier defects, uncontrolled inflammation, tissue fibrosis, and neural dysfunction, making comprehensive repair difficult with single treatment modalities. Based on cutting-edge technologies, four major pathways are constructed: cell therapy, molecular drug therapy, tissue engineering therapy, and combination therapy, achieving a full-chain treatment of "targeted regulation – tissue repair – functional remodeling".

### Bio-hybrid systems therapy: design and construction from engineered bacteria to smart cells

We foresee that the deep integration of synthetic biology and nanotechnology will give rise to a new generation of bio-hybrid therapeutic systems. These systems are expected to bring about a paradigm revolution in IC treatment through precise regulation of the local microenvironment and targeted repair of damaged tissue.

Engineered Bacterial Therapeutic System: *Escherichia coli* Nissle 1917 (*EcN*), modified by synthetic biology, can be engineered as a programmable "sense-and-respond" biological computer for localized therapeutic delivery [16]. Given that the bladder and intestine are both mucosal tissues (both possessing epithelial barrier structures and mucosal immune microenvironments), and *EcN* has been shown to transiently colonize the urinary tract, its mucosal repair mechanisms in inflammatory bowel disease could be translatable to the bladder inflammation context, providing a theoretical basis for subsequent designs.

A feasible design involves constructing a pH-responsive genetic circuit based on the principle of pH-inducible gene expression (pHLux/pR promoter system). In this system, the pHLux promoter can be activated by protonated transcription factors in an acidic environment (pH < 5.5), thereby initiating downstream gene expression. Similarly, this could enable engineered bacteria to automatically initiate the expression of anti-inflammatory factors IL-10 and TGF-β under the low pH conditions (5.5) of an IC patient's bladder [17].

Theoretically, this system could significantly increase the local concentration of IL-10 and other factors (referencing enrichment efficiency in gut models and effectively downregulate pro-inflammatory cytokine levels like TNF-α and IL-6 [16].Furthermore, a Quorum Sensing regulatory module could be introduced, where upon reaching a preset bacterial density threshold, a secondary genetic circuit is automatically triggered, driving the engineered bacteria to express hyaluronic acid synthase (HAS2), thereby directly participating in the repair of the bladder GAGs layer [18]. This closed-loop design of "sensing-response-treatment". In future animal model validations, it might significantly improve urinary frequency symptoms and bladder mucosal integrity.Current validation gaps and challenges are outlined in Table 2.

To date, this platform remains a conceptual design; no experimental validation in bladder models or IC/BPS has been reported. The following description outlines a theoretical framework for future investigation.

Optogenetically-Controlled Macrophage-Nano Hybrid System: Targeting the submucosal nerve fiber hyperplasia associated with IC, based on the macrophage membrane-coated MOF nanoparticles [19], we conceptualize an engineered macrophage membrane-coated Metal–Organic Framework platform (MOFs@iMAC). The design concept of this platform is as follows: Transfect the photosensitive protein Channelrhodopsin-2 (ChR2) into M2-type macrophages, and use their membrane structures to encapsulate a zirconium-based MOF (UiO-66-NH_2_) loaded with Nerve Growth Factor (NGF) and KGF-1 — UiO-66-NH_2_, as a typical stable zirconium-based MOF, possesses a porous structure with drug loading potential [20].

The above research provides a theoretical basis for the concept that under near-infrared light irradiation at a specific wavelength (e.g., 808 nm), the membrane potential change triggered by ChR2 might promote the opening of MOF pores, achieving on-demand drug release.

This concept has multiple potential advantages: (1) The engineered macrophage membrane could confer inflammatory targeting ability to the nanoparticles; (2) Near-infrared optogenetic control could enable spatiotemporally precise control of drug delivery; (3) The Zn^2+^ slowly released from the MOF framework might help inhibit abnormal firing of bladder sensory nerves. If this strategy is validated in future preclinical studies, it might significantly increase the bladder pain threshold and reduce mast cell infiltration.

### Molecular drug therapy: targeted molecular regulation based on intelligent delivery systems

Molecular drug therapy for IC leverages intelligent delivery systems to achieve precise, long-acting, and synergistic drug release, thereby overcoming the limitations of poor tissue penetration and off-target effects inherent to conventional treatments [21]. Design Concept for Environmentally-Responsive DNA Nanomachines: Traditional drugs face challenges of short residence time and poor tissue penetration within the bladder. One potential strategy involves utilizing the pH fluctuations of the IC pathological microenvironment (4.5–6.5), a well-documented pathological hallmark [21], as the intelligent trigger condition. For example, design a Topological DNA Nanocage (TDN) that remains closed at normal physiological pH (7.4) but unfolds in the acidic bladder environment, exposing targeting peptides (PLZ4) and epigenetic drugs (Trichostatin A/DAC) [22, 23]. The TDN leverages its topological structure to simultaneously achieve pH responsiveness, targeted recognition, and dual-drug loading design rationality; Furthermore, the synergy between photodynamic therapy and gene therapy in enhancing efficacy in other systems provides theoretical support for the TDN system's combined strategy of simultaneously alleviating fibrosis and regulating epigenetics [24].

The innovation of this system might lie in: its dynamic programmability allows setting the pH trigger threshold by adjusting the GC base ratio; its synergistic therapeutic potential can simultaneously alleviate fibrosis and regulate epigenetics; through surface modification with polyethylene glycol-dopamine copolymers, it might be possible to extend the intravesical residence time severalfold. Stability and penetration limitations are detailed in Table 2

Physical Strategy of Magnetoelectric Nanorobots for Enhanced Tissue Penetration (Conceptual): To overcome the limitation of drug penetration by the bladder epithelial barrier in IC patients, magnetoelectric nanoparticles represent a potential conceptual strategy. Core–shell nanoparticles comprising a magnetostrictive core (e.g., Fe_3_O_4_) and a piezoelectric shell (e.g., BaTiO_3_) can generate localized electric fields under alternating magnetic field stimulation. While the defect and interfacial structure of such Fe_3_O_4_/BaTiO_3_ bilayers has been characterized at the nanoscale [25], their application in enhancing drug transport across the bladder epithelium remains purely theoretical at this stage. It is hypothesized that the combined magnetothermal effect and piezoelectric surface charge could transiently increase membrane permeability by modulating tight junctions or activating mechanosensitive ion channels. However, no experimental data currently exist validating this permeability enhancement in bladder epithelial models, and quantitative claims regarding the magnitude of such enhancement require rigorous validation in urological tissue contexts before clinical translation can be considered. (See Table 2 for current validation status and key translational gaps.)

Looking ahead to the next five years, these technologies might catalyze the formation of a new field of 'Precision Bladder Medicine'. For example, real-time monitoring of treatment response through urine tests could enable dynamic adjustment of bacterial activity or light parameters. However, bio-hybrid systems still face challenges: long-term colonization safety of engineered bacteria, large-scale GMP production of nanomaterials, and cost control for individualized therapy. With advances in organoid-on-a-chip and AI predictive models, these obstacles are expected to be gradually overcome, ultimately rewriting the treatment landscape for IC.

### Tissue engineering therapy: revolutionary breakthroughs of biomimetic manufacturing and organoid intelligence in IC treatment

Digital twin technology is the carrier for medical-engineering integration to empower integrated diagnosis and treatment. We foresee that the deep integration of biomimetic manufacturing and organoid intelligence technologies will bring a paradigm shift to IC treatment. It is expected that through the organic combination of intelligent biomaterial systems and digital regulation strategies, a full chain new therapeutic strategy from mucosal repair to functional remodeling will be constructed [26].

Biomimetic Intelligent Hydrogel Repair System: Targeting the core pathological features of impaired elastic function of the bladder wall layer and persistent chronic inflammation in IC patients, we propose the development of a perfusable biomimetic intelligent hydrogel system as a new future pathway for intravesical therapy of IC [27]. The design of this system centers on environmentally responsive biomaterials, such as functionalized hyaluronic acid-gelatin composite hydrogels [28]. The innovation of this system lies in two intelligent design features. First, it exhibits dynamic mechanical property adaptation: incorporation of thermosensitive polymer segments (e.g., poly(N-isopropylacrylamide)) or utilization of the thermosensitive mechanism of HA-Gel enables the hydrogel to remain liquid at 15–37.5 °C for easy instillation, yet solidify within 1 min upon exposure to body temperature (∼37.5 °C) after administration, forming a viscoelastic three-dimensional networkv [29]. Its theoretical elastic modulus could be designed to match the mechanical properties of native bladder tissue, while the solid state could prolong the material's residence time in the bladder, solving the problem of short residence time of traditional drug instillation, thereby providing compliance support during filling and emptying cycles and alleviating pain caused by tissue hardening [29].

Inflammatory Microenvironment Response and Regulation: Embedding pH-sensitive nanocapsules within the hydrogel network, conceptualized to intelligently release loaded IL-10 plasmids or small molecule anti-inflammatory drugs in the acidic urine environment associated with IC. Simultaneously, the hydrogel backbone itself could be covalently grafted with cell adhesion peptides (e.g., RGD peptides), aiming to competitively inhibit excessive adhesion of inflammatory cells and promote the natural defense mechanisms of the bladder mucosa. Theoretically, this system could potentially form a dynamic "inflammation regulation—tissue protection" microenvironment locally at the lesion site. Mechanical fatigue and long-term biocompatibility data are summarized in Table 2.

Conceptualization of an Organoid-on-a-Chip System Integrating Sensing and Regulatory Functions. We envision an organoid-on-a-chip system integrated with multi-modal sensing functions to address the disordered bladder tissue microenvironment and functional abnormalities in IC. This system would enable dynamic monitoring and precise regulation of the tissue repair process [30].

The core design of this system is based on the deep integration of bladder-derived organoids and miniature sensing elements. We propose the following innovative architecture: We propose an innovative architecture that references the core structural logic of Vascularized Organoid-on-a-Chip (VOoC) systems [31]. VOoC research emphasizes the necessity to combine efficient analysis techniques like deep learning for processing large-scale imaging data [31]. This logic supports our requirement for rapid interpretation of complex physiological data from bladder organoids. Furthermore, VOoC designs that support cell growth within hydrogel regions containing specific growth factors and maintain microenvironmental homeostasis via controlled fluid perfusion offer valuable insights. We can reference their technical approaches for optimizing hydrogel biocompatibility and precisely regulating fluidic parameters [32]. Building on these microfluidic integration strategies, we further propose a multi-parameter physiological signal monitoring platform. This platform integrates a high-density flexible microelectrode array, which could potentially enable non-invasive recording of spontaneous potentials (expected frequency range 0.5–3 Hz) and action potential conduction patterns of smooth muscle cells within the organoid [33]. Theoretically, by analyzing characteristic parameters of the electrical signals, it might be possible to early identify neuromuscular functional abnormalities associated with IC.

Dynamic Microenvironment Regulation Module: The microfluidic network embedded in the chip allows precise control of the local biochemical environment [34]. We integrate ultra-miniature oxygen-sensitive fluorescent probes (e.g., platinum porphyrin complexes) and pH sensing units within the system for continuous monitoring of oxygen partial pressure and pH dynamics at the tissue level. When the system detects pathological signals such as hypoxia (e.g., pO_2_ < 15 mmHg) or increased acidity, it can automatically trigger micro-pumps to release specific repair factors (e.g., VEGF, FGF2), aiming to improve local microcirculation.

Closed-Loop Feedback Therapeutic Potential: Further, we envision coupling this system with the aforementioned intelligent hydrogel technology to construct a closed-loop treatment system of sensing-feedback-intervention. Real-time physiological data obtained by the organoid-on-a-chip could be wirelessly transmitted to an external analysis system, which then guides the adjustment of the drug release pattern of the intelligent hydrogel, achieving personalized dynamic management of the bladder repair process. This system fills the critical gap in dynamic, non-invasive tools for monitoring IC bladder tissue repair and enables real-time assessment of pathological signaling and drug response at the organoid level*.*

Digital Twin Pathway for IC Patient-Specific ECM: The ECM of the bladder in IC patients is characterized by an inverted collagen III/I ratio and elevated fibronectin fragmentation. [35]. We conceptualize constructing a nanoscale ECM topology database by integrating second harmonic generation microscopy and atomic force microscopy, and utilizing AI algorithms to parse its complex fiber orientation and mechanical gradients [36]. This digital twin model could guide the preparation of highly biomimetic nanofiber scaffolds [37]. Through technologies like dynamic electric field regulation, it might be possible to achieve a gradient distribution of collagen to match the patient's specific pathological features. Additionally, using atomic force microscopy data to train generative adversarial networks could predict local mechanical properties of the ECM and feedback to adjust the crosslinking density of the scaffold, achieving precise adaptation of mechanical properties.

We further propose the development of a photo-responsive hydrogel-nanorobot composite for spatiotemporally precise regulation of the extracellular matrix (ECM). The feasibility of this system is substantiated by cutting-edge research prototypes: for instance, integrated "sensing-actuation" closed-loop microrobot systems incorporating implantable sensors and external trigger actuators have been documented. These systems enable real-time monitoring of tissue microenvironment parameters (e.g., pH, inflammatory factors) and initiate functional molecule release via near-infrared light or focused ultrasound upon detecting abnormalities. Their core control logic demonstrates high compatibility with our system's "quantum dot sensor-gold nanorod-mediated matrix metalloproteinase release" architecture. Furthermore, the efficacy of their acoustic/magnetic dual-driven targeted delivery has been validated at the in vivo level, establishing a robust technical framework for intelligent ECM remodeling systems [38, 39]. In our proposed system, implantable quantum dot sensors would continuously monitor critical indicators, including pH and IL-6 concentrations, with data transmitted wirelessly via miniature devices. The positive correlation between IL-6 concentration dynamics and ECM fibrosis progression validates its rationale as a core monitoring signal, ensuring accurate detection of ECM abnormalities. Upon identification of fibrotic signals, near-infrared light activation would mediate controlled release of matrix metalloproteinases from gold nanorods to degrade pathological collagen deposits [40]. Regarding clinical validation and digital twin iteration, should this strategy progress to clinical testing phases, future prospective studies may demonstrate its potential for enhancing bladder compliance and ameliorating urinary urgency symptoms. Concurrently, postoperative acquisition of real-world ECM data feedback into the digital twin system would drive continuous iterative optimization of predictive models, thereby establishing the foundation for truly personalized dynamic treatment adjustments [41].

### Combination therapy: synergistic and complementary pathways of the three single-modality therapies

The complex pathological mechanisms underlying IC determine that monotherapy is unlikely to achieve comprehensive cure. Combination therapy, through pairwise synergies among cellular therapy, molecular pharmacotherapy, and tissue engineering approaches, demonstrates enhanced therapeutic efficacy-particularly suitable for refractory IC cases.

#### Synergy of engineered bacteria and intelligent hydrogels: enhanced colonization and sustained release

Although engineered *EcN* can target inflammatory regions, its retention time in flowing urine remains a challenge. If *EcN* is combined with a thermo/pH dual-responsive hydrogel (e.g., hyaluronic acid-gelatin system) for co-instillation, a practical solution could be formed. Engineered EcN has demonstrated the capacity for localized delivery of anti-inflammatory cytokines in mucosal tissues  [16, 17], yet its rapid clearance from the bladder due to urinary flow remains a major obstacle. The hydrogel rapidly gels at body temperature, providing physical anchorage for engineered bacteria and preventing rapid clearance [42]. In the acidic bladder microenvironment, it degrades to enable sustained release of IL-10 plasmid, synergizing with bacteria-secreted anti-inflammatory factors [42]. While this combinatorial strategy has not yet been validated in preclinical IC/BPS models, it represents a rational integration of two independently established technologies.This combination, starting from practicality, uses clinically feasible instillation methods and solves the stability issue of live bacteria therapy through material assistance. Functional hydrogels can effectively prolong the action time of drugs in the bladder and improve bladder compliance.

#### Near-infrared light-responsive drug release system: achieving spatiotemporally precise regulation of molecular drugs

To address the challenges of the focal distribution of IC lesions and the easy washout of traditional instilled drugs by urine or their non-specific diffusion to normal tissues, we propose constructing a near-infrared light (NIR) responsive intelligent delivery system based on upconversion nanoparticles (UCNPs). This system aims to achieve "on-demand release" and "spatial enrichment" of molecular drugs, thereby accomplishing spatiotemporally precise therapeutic intervention.

Its technical core lies in using core–shell UCNPs to convert 980 nm infrared light to ultraviolet or visible light. These UCNPs can be further surface-loaded with photo-responsive polymers and therapeutic molecules (e.g., anti-inflammatory factor IL-10, GAGs precursor hyaluronic acid) [43, 44]. When external NIR light precisely irradiates the bladder lesion area, the UV light generated in situ by the UCNPs will trigger reversible photo-isomerization, causing conformational changes or depolymerization of the polymer, thus precisely controlling the release kinetics of the encapsulated drugs [45]. By adjusting the irradiation parameters of the NIR light (e.g., intensity, duration, interval), it can flexibly match the treatment needs of different pathological stages of IC—achieving rapid drug release in the acute inflammatory phase to quickly control symptoms, and switching to sustained release in the chronic repair phase to promote long-term tissue recovery [45, 46].

NIR light possesses excellent tissue penetration ability, effectively covering the full thickness of the bladder wall, overcoming the technical bottleneck of insufficient penetration depth of UV/visible light, ensuring that deep lesions also receive sufficient drugs. Secondly, this strategy achieves "light-controlled localized release" of drugs, strictly confining drug activity to the illuminated area, greatly reducing non-specific exposure of the drug to normal bladder tissue and the potential chemical irritation it may cause. Furthermore, the inherent upconversion luminescence properties of UCNPs themselves can be used for real-time tracking of drug distribution within the bladder, providing visual feedback for the precise targeting of NIR irradiation, ensuring therapeutic energy covers all target lesions (e.g., Hunner's ulcer areas or specific GAGs defect areas), thereby comprehensively enhancing the precision and reliability of treatment.

#### Integration of nanodrugs and tissue engineering scaffolds: achieving simultaneous structural and functional repair

**Fig. 2:**
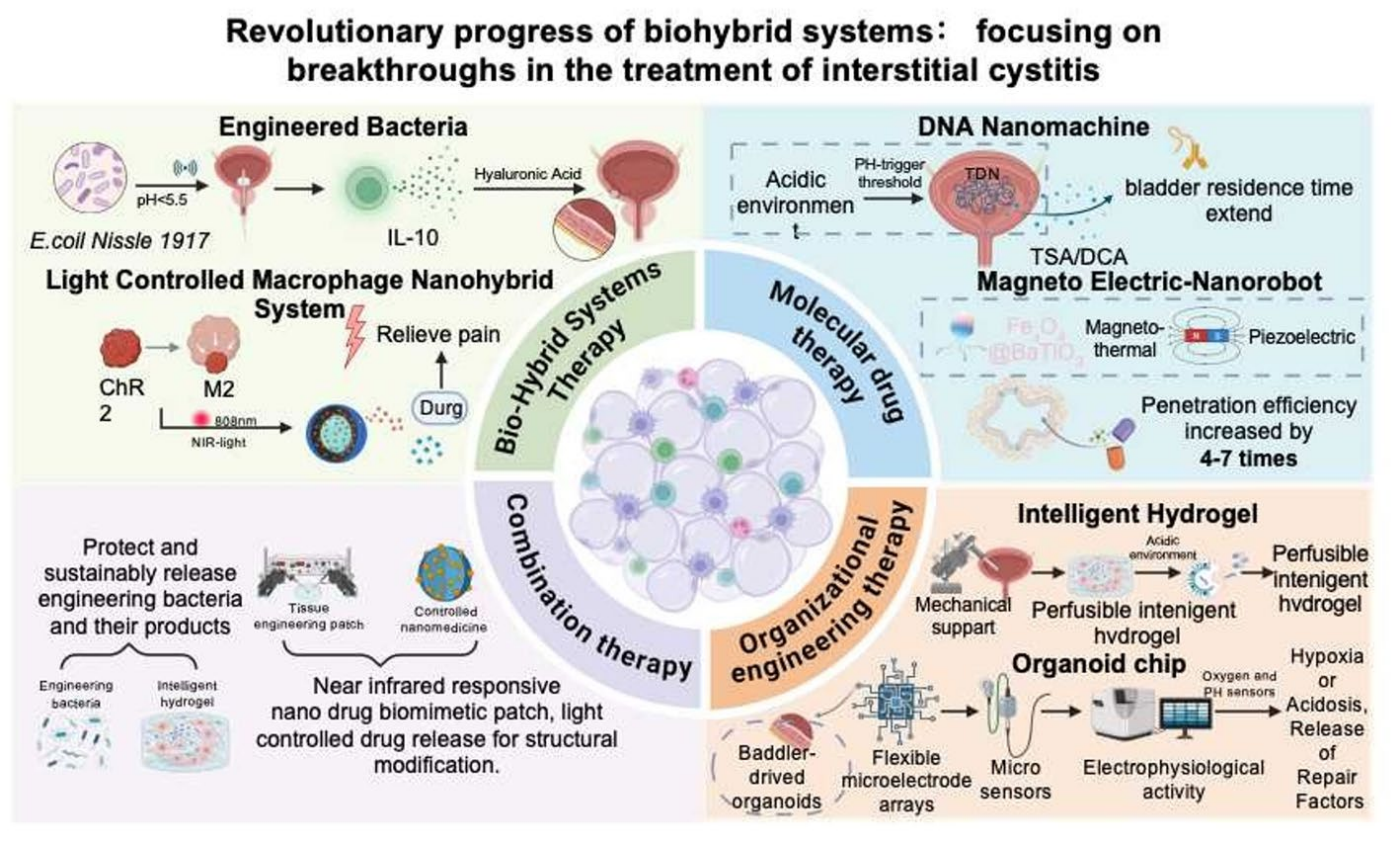
Schematic diagram of therapeutic technologies for IC

For patients presenting with Hunner's lesions or severe mucosal defects, structural reconstruction cannot be adequately achieved through intravesical drug instillation alone. A promising therapeutic approach involves integrating topology-controlled DNA nanocages (TDN) loaded with epigenetic modulators with 4D-bioprinted biomimetic patches [22, 26]. The patch serves dual functions: providing mechanical support to guide tissue regeneration, while its porous architecture acts as a reservoir for nanodrugs [37]. The pH-responsive characteristics of TDN ensure preferential drug release within the acidic microenvironment of the pathological sites [22]. This combinatorial strategy offers clinical practicality by simultaneously addressing two fundamental challenges—tissue defect restoration and local microenvironment modulation—through a single patch implantation procedure. Furthermore,DNMT/HDAC inhibition may potentially induce immunogenic neoantigens, thereby reprogramming the local immune microenvironment in a manner synergistic with the requirements of tissue regeneration processes [10] (Fig. 2).

## Neuromodulation: conceptions on future pathways for the cross-domain integration of flexible electronics and brain-computer interfaces

We anticipate that the cross-domain integration of flexible electronics and brain-computer interface (BCI) technologies will open new dimensions for the precise diagnosis and treatment of IC, particularly demonstrating transformative potential in the field of neuromodulation [47]. The following sections explore potential future development paths from two perspectives: implantable neuromodulation systems and brain-bladder bidirectional interfaces.

### Implantable neuromodulation systems and energy harvesting

Closed-Loop Regulation Potential of Graphene-Based Multimodal Flexible Electrode Arrays (GM-FEA): Targeting the key pathological feature of abnormal nerve root signal conduction in IC patients, where traditional rigid electrodes are limited by mechanical mismatch, we propose the future use of graphene-based multimodal flexible electrode arrays (GM-FEA). Their flexible substrate theoretically allows for superior conformance to the curved anatomy of nerve roots, achieving more stable and reliable interface contact. This conception is supported by relevant research. A study involving accelerated aging tests at 87 °C, equivalent to approximately 960 days in vivo, demonstrated that graphene electrodes on PET substrates remained fully functional across all channels. Their average impedance stabilized around 585 kΩ, and during 45-day in vivo experiments, they exhibited low noise levels, enabling high-fidelity recording of neural signals. In contrast, electrodes on SU-8 substrates typically failed within 1–2 weeks due to cracking, underscoring the potential of PET substrates to ensure long-term implant stability for GM-FEA [48]. Additionally, the array's inherent high optical transparency facilitates the simultaneous acquisition of multi-channel neural electrical signals and optical signals (e.g., for optogenetics or calcium imaging) which could be instrumental in deciphering IC-related abnormal neural discharge patterns [49]. Building on this foundation, we conceive a closed-loop regulation system. Embedded machine learning algorithms would analyze neural electrical signal features in real-time. Upon identifying signature patterns associated with pain or bladder dysfunction, the system could automatically trigger precisely calibrated electrical stimulation parameters designed to inhibit the conduction of pathological afferent signals. GM-FEA achieve stable signal acquisition by conforming to the anatomical structure of nerves, and block pain conduction in combination with closed-loop electrical stimulation. Realization of this technical pathway could establish a new paradigm for neuromodulation therapy in IC.Current implantation and recording limitations are provided in Table 2.

Energy Harvesting Concept via Self-Powered Piezoelectric Nanogenerators (PENG): Addressing the challenge of providing long-term power for implantable electronic devices, we propose utilizing piezoelectric nanogenerators (PENG) to harvest mechanical energy generated by the natural contractions and movements of the bladder wall [50]. This concept leverages the intrinsic mechano-electric conversion characteristics of piezoelectric materials (e.g., BaTiO_3_ nanowire arrays), transforming bladder mechanical energy into electrical energy to sustainably power the GM-FEA-based closed-loop neuromodulation system described above. The PENG is therefore not a standalone alternative, but an integrated energy module within a unified peripheral neuromodulation platform. This technical approach shares core principles with existing research on implantable triboelectric nanogenerators (TENG): such TENGs have successfully harvested and converted dynamic mechanical energy from organs like the heart and muscles using bio-compatible, and sometimes biodegradable, materials, offering valuable insights regarding material selection and energy harvesting strategies from autonomous organ motions for PENG development [51]. PENGs convert the mechanical energy of bladder contraction into electrical energy to power implantable neuromodulation devices. The electrical signals generated by PENG themselves might exert a modulatory influence on local neural activity, potentially indirectly affecting the inflammatory state within the bladder wall. This characteristic, alongside the demonstrated capability of TENGs to monitor physiological signals (e.g., recording apex cardiograms) beyond mere energy harvesting, reveals a common theme of "energy-function synergy" across different nanogenerator technology routes [51], providing cross-platform theoretical support for potential ancillary benefits in IC treatment.PENGs have been validated for energy harvesting in cardiovascular and gastrointestinal systems, but their application in the bladder under urodynamic conditions has not yet been tested [50].Validation status in urodynamic conditions is noted in Table 2.

### Innovative directions for brain-bladder bidirectional interfaces

Conception of Central Regulation via Neuromorphic Computing Chips: Central sensitization plays a key role in IC symptom persistence, as evidenced by functional MRI alterations in pain-related brain regions and quantitative sensory testing demonstrating segmental hyperalgesia [52]. Targeting this maladaptive 'pain memory' within the central nervous system, we propose a neuromodulation concept based on memristor-equipped neuromorphic computing chips. This proposal is grounded in sound technical principles. The core property of memristors lies in their reconfigurable resistance states, which can precisely emulate the synaptic weight changes underlying learning and memory in biological neural networks, exhibiting a high degree of congruence with the mechanisms of neural plasticity. This provides a crucial foundation for hardware-based simulation of neural plasticity and subsequent intervention in maladaptive pain pathways [53].

Further research has demonstrated that memristor-based neuromorphic systems possess the capability for real-time analysis of neural electrical activity and identification of aberrant signal patterns. For instance, such systems have achieved successful detection of neuronal action potentials and recognition of specific brain functional states, thereby establishing the technical feasibility for a system to identify IC-related abnormal neural activity patterns and potentially predict precursors to pain episodes. Memristor-based neuromorphic computing chips predict IC pain attacks and trigger precise stimulation by simulating synaptic plasticity. Additionally, the low power consumption characteristics of memristors and their favorable compatibility with conventional CMOS fabrication processes make them particularly suitable for implantable medical device applications [1]. Experience gained from their use in closed-loop neuromodulation systems, such as the precise modulation mechanisms employed in closed-loop BCIs, also lends practical credence to the concept of triggering targeted neural stimulation in our proposed system [1]. Such a chip could potentially mimic the plasticity inherent in the nervous system. By continuously analyzing the features of central nervous system (CNS) electrical activity, it could learn to identify patterns characteristic of IC. Upon predicting an impending pain episode, it could trigger precisely timed and located neuromodulatory stimuli. In the long term, such a system might gradually reverse the maladaptive changes in pain pathways through targeted modulation of neural plasticity, offering a novel approach for the long-term management of IC.

Exploration of Technical Pathways for Targeted Neuromodulation. For the specific neural circuit dysfunctions implicated in IC, we conceive a targeted modulation strategy that combines gene therapy with optical neuromodulation. This involves local delivery of genes encoding light-sensitive proteins (e.g., channelrhodopsins) to the bladder wall or relevant neural pathways, potentially using viral vectors. To overcome the limited penetration of visible light, NIR-responsive nanoparticles (e.g., upconversion nanoparticles) could be employed to act as transducers, converting deeply penetrating NIR light into visible wavelengths that activate the opsins. Guided by real-time functional magnetic resonance imaging (fMRI) or other advanced neuroimaging techniques, this approach could theoretically enable precise manipulation of activity in specific neural nuclei involved in bladder sensation and function, thereby intervening in aberrant signal transmission and normalizing bladder control. Optogenetic nanoprobes (AAV-ChR2@UCNPs) activate photosensitive proteins by converting near-infrared light (to visible light) to regulate the bladder sensory nerve pathway.

## Future vision and outlook: the systematic medicine revolution of material intelligence agents

The deep coupling of materials science and clinical needs is driving the evolution of IC diagnosis and treatment towards "Cross-Scale Intelligent Systems Medicine". Systems based on AI Material Agents (AIMAs) integrate nanosensors, quantum computing, and micro-nano robots, achieving multi-level precise intervention from molecules to organs, and constructing an integrated diagnostic and therapeutic closed loop. Nanosensor networks can monitor bladder microenvironment inflammatory factors, barrier integrity, and fibrosis levels in real-time, with data transmitted via 5G/6G to cloud AI platforms, building dynamic inflammation scoring models [54] Ultrafast manipulation. Quantum annealing algorithms can rapidly optimize drug combinations and administration strategies, avoiding side effects, and significantly enhancing treatment personalization. Micro-nano robots could theoretically execute targeted tasks, such as clearing inflammatory cells, repairing the epithelial barrier, and regulating neural activity, thereby potentially forming a 'sensing-decision-execution' closed loop—nanosensors collect data, 5G transmission to the cloud, quantum computing rapidly optimizes treatment plan, signals feedback to micro-nano robots for targeted task execution, achieving a positive feedback cycle from local repair to functional recovery.

Digital twin technology serves as the carrier for medical-engineering integration to enable integrated diagnosis and treatment. It integrates in vitro experimental data from organoid-on-a-chip, multi-modal radiomics, and epigenetic maps to construct a virtual digital twin bladder model. This model can simulate the impact of different intervention strategies on disease progression, guiding precise treatments like CRISPR epigenetic editing and controlled drug release. The system possesses self-iteration capability, continuously optimizing prediction accuracy with real-world data feedback, ultimately achieving dynamic adjustment of integrated diagnosis and treatment tailored to patient-specific strategy. Building a bladder digital twin requires multimodal data—imaging, urodynamics, molecular profiles, and real-time sensor outputs. The feasibility of such an approach is supported by established digital twin frameworks in other medical fields; for instance, Coorey et al. outlined the use of digital twins for personalized treatment planning in cardiology, providing a transferable methodological blueprint for chronic inflammatory diseases like IC [55].

**Fig. 3:**
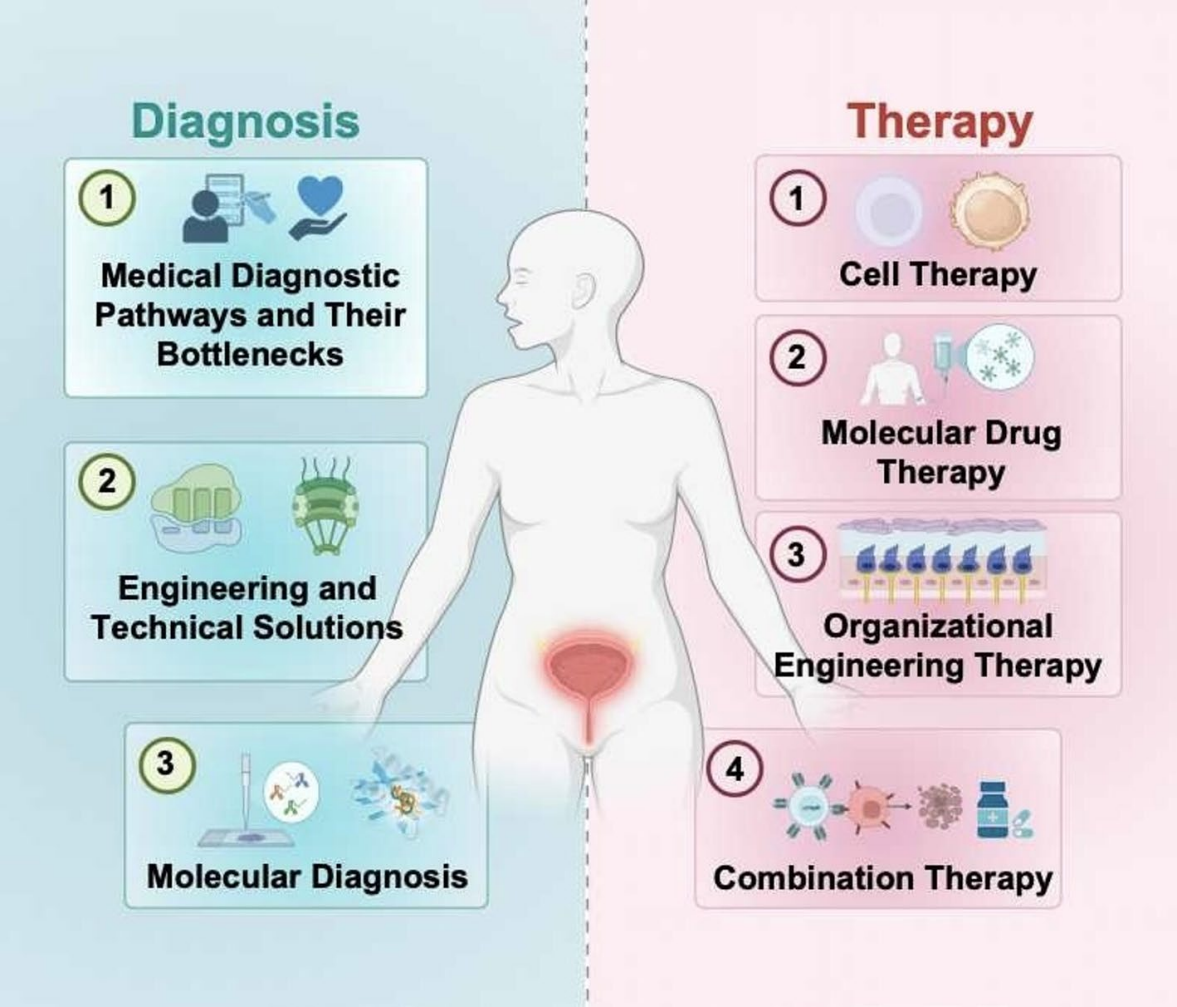
Schematic diagram of multi-dimensional integrated diagnosis and treatment system for IC

Materials science, supported by medical-engineering integration, is reshaping the entire paradigm chain of IC diagnosis and treatment: from quantum sensing at the molecular scale, to synthetic biology regulation at the cellular scale, to 4D bioprinting at the organ scale, finally achieving integrated diagnosis and treatment at the systems medicine level. Pending rigorous preclinical and clinical validation at each level of integration, the conceptual framework described herein may contribute to advancing IC management toward more predictive, preventive, personalized, and participatory strategies in the coming decade. It is recommended to prioritize the development of biocompatible quantum materials, establish standardized databases for IC digital twins, and formulate ethical guidelines for medical-engineering integration, collectively advancing the transformation of IC precision management from concept to clinical practice. (Fig. 3).

## Challenges and coping strategies

Despite the paradigm-shifting potential demonstrated by intelligent material systems in IC diagnosis and treatment, their clinical translation faces substantial challenges. The primary concern lies in biocompatibility and long-term safety. The genetic stability of engineered bacteria, in vivo accumulation behavior of nanomaterials, and long-term biocompatibility of implantable devices are critical determinants of clinical applicability. Countermeasures include developing bioinspired coatings and biodegradable materials, alongside designing stringent controllable safety switches for active components. Secondly, manufacturing processes and regulatory frameworks present industrial bottlenecks. Standardized and compliant production of complex systems poses significant challenges, while existing regulatory systems struggle to accommodate such cross-disciplinary products. This necessitates advancing modular and automated advanced manufacturing, coupled with preemptive development of risk-based adaptive regulatory pathways through industry-academia-regulatory collaboration. Furthermore, while personalized treatment regimens offer precision, they encounter challenges in cost and accessibility. A tiered strategy is recommended: initial patient stratification via biomarkers, followed by matching with appropriately-tiered intelligent solutions to optimize resource allocation. Concurrently, leveraging automated platforms and open-source models can reduce costs, supplemented by parallel health economic evaluations. Finally, neural interface and digital twin technologies raise novel ethical and data security concerns. Dedicated ethical review mechanisms must be established, incorporating advanced privacy-preserving computational technologies to ensure patient data security and autonomous decision-making rights. Only through sustained interdisciplinary collaborative innovation can these challenges be systematically addressed. enabling the robust translation of these cutting-edge concepts into clinical practice for ultimate patient benefit [56].

## Data Availability

No datasets were generated or analysed during the current study.
